# Risk factors, prediction model, and prognosis analysis of myocardial injury after acute upper gastrointestinal bleeding

**DOI:** 10.3389/fcvm.2022.1041062

**Published:** 2022-12-08

**Authors:** Junjun Hao, Peizhu Dang, Xingpu Quan, Zexuan Chen, Guiyun Zhang, Hui Liu, Tao Shi, Yang Yan

**Affiliations:** ^1^Department of Cardiovascular Surgery, The First Affiliated Hospital of Xi’an Jiaotong University, Xi’an, China; ^2^Department of Cardiovascular Medicine, The First Affiliated Hospital of Xi’an Jiaotong University, Xi’an, China; ^3^Department of Gastroenterology, The First Affiliated Hospital of Xi’an Jiaotong University, Xi’an, China; ^4^The Biobank of The First Affiliated Hospital of Xi’an Jiaotong University, Xi’an, China

**Keywords:** prediction model, acute upper gastrointestinal bleeding, myocardial injury, cancer, risk factor

## Abstract

**Background:**

Cardiovascular complications in patients with acute upper gastrointestinal bleeding (AUGIB) have been associated with a high-risk of subsequent adverse consequences. This study aimed to analyze the risk factors for myocardial injury in AUGIB patients, predict the risk of myocardial injury, and explore the clinical prognosis and influencing factors in AUGIB patients with myocardial injury.

**Materials and methods:**

A retrospective case-control study based on AUGIB patients in the First Affiliated Hospital of Xi’an Jiaotong University from 2016 to 2020 was performed. We divided the enrolled patients into a myocardial injury group and a control group according to whether they developed myocardial injury. The variables significant in the univariate analysis were subjected to binary logistic regression for risk factor analysis and were used to establish a nomogram for predicting myocardial injury. In addition, logistic regression analysis was performed to better understand the risk factors for in-hospital mortality after myocardial injury.

**Result:**

Of the 989 AUGIB patients enrolled, 10.2% (101/989) developed myocardial injury. Logistic regression analysis showed that the strong predictors of myocardial injury were a history of hypertension (OR: 4.252, 95% CI: 1.149–15.730, *P* = 0.030), blood urea nitrogen (BUN) (OR: 1.159, 95% CI: 1.026–1.309, *P* = 0.018) and left ventricular ejection fraction (LVEF) <68% (OR: 3.667, 95% CI: 1.085–12.398, *P* = 0.037). The patients with a tumor history (digestive system tumors and non-digestive system tumors) had no significant difference between the myocardial injury group and the control group (*P* = 0.246). A prognostic nomogram model was established based on these factors with an area under the receiver operator characteristic curve of 0.823 (95% CI: 0.730–0.916). The patients with myocardial injury had a much higher in-hospital mortality rate (10.9% vs. 2.0%, *P* < 0.001), and an elevated D-dimer level was related to in-hospital mortality among the AUGIB patients with myocardial injury (OR: 1.273, 95% CI: 1.085–1.494, *P* = 0.003).

**Conclusion:**

A history of hypertension, renal dysfunction, and cardiac function with LVEF <68% were strong predictors of myocardial injury. Coagulopathy was found to be associated with poor prognosis in AUGIB patients with myocardial injury.

## Introduction

Acute upper gastrointestinal bleeding (AUGIB) is a common, life-threatening clinical emergency with an incidence of 84–160/100,000 per year (1–3). Despite significant progress in the treatment of AUGIB in recent years, including the primary application of endoscopic therapy, proton pump inhibition, vasoactive pharmacotherapies and prophylactic antibiotics (4), it still imposes considerable morbidity, mortality and health economic burdens on patients, and these factors have remained stable for the past 30–40 years (5). Previous studies have shown that in-hospital cardiovascular events may lead to increased mortality in AUGIB patients, with most of these cardiovascular events manifesting as myocardial injury.

Non-fatal bleeding not only causes great discomfort, but also leads to more harmful, even life-threatening, ischemic events (6). Several hypotheses exist to explain the link between AUGIB and myocardial injury. First, gastrointestinal bleeding, especially with massive blood loss, may precipitate myocardial injury from hypovolemia, hemodynamic compromise, and myocardial hypoperfusion (7). Additionally, bleeding leads to reduced renal perfusion, which results in significantly higher levels of myocardial oxidative stress and inflammation (8). Moreover, some patients with AUGIB could develop acute coronary syndrome (ACS), which increases the additional risk of intensive care unit (ICU) admission and accounts for up to 30% of in-hospital deaths (9–11). Therefore, myocardial injury after AUGIB has attracted increasing attention as a significant international public health problem. However, little data are known about the potential risk of myocardial injury in patients with AUGIB. This study aimed to analyze the risk factors for myocardial injury after AUGIB by statistical analysis and prediction model construction and to explore the association between myocardial injury and poor in-hospital prognosis.

## Materials and methods

### Ethical approval and consent

This single-center, retrospective study was approved by the Ethics Committee of The First Affiliated Hospital of Xi’an Jiaotong University (no. XJTU1AF2022LSK-301), and informed consents were obtained from the patients. This study was conducted in accordance with the relevant regulations and the guidelines of the Declaration of Helsinki.

### Participants

**FIGURE 1:**
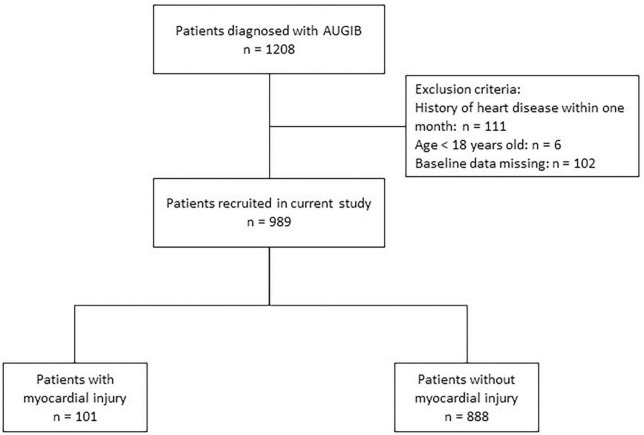
Flowchart of the study. AUGIB, acute upper gastrointestinal bleeding.

Patients hospitalized for AUGIB from January 2016 to December 2020 at the First Affiliated Hospital of Xi’an Jiaotong University were consecutively enrolled. AUGIB was defined as clinical events with the chief complaint of blood vomitus or tarry stool passage or the presence of blood in the upper gastrointestinal tract on endoscopic evaluation (12). The exclusion criteria included: (1) patients with a history of heart disease (including abnormalities in the structure and function of the heart such as coronary artery disease, arrhythmias, heart failure or heart-valve defect) within 1 month; (2) age younger than 18 years; and (3) missing baseline data. Myocardial injury was defined as the presence of any of the following: ST-T changes (horizontal or arch-back-up elevation of ST-segment ≥0.1 mV or with T-wave elevation) (13) in two consecutive leads on electrocardiogram (ECG); elevated troponin T (TnT) >0.014 ng/mL or troponin I (TnI) >26.2 pg/mL (14, 15). The study flowchart is shown in Figure 1. We excluded patients with a history of heart disease within 1 month to ensure that the myocardial injury occurred after AUGIB, thus avoiding the bias caused by myocardial injury due to heart attack.

### Clinical data collection

All of the patients’ clinical data were collected from their medical records provided by the biobank. The data were collected from patients at the time of admission and included baseline demographics [age, sex, medical history of hypertension, diabetes, and chronic kidney disease (CKD)], laboratory examination [complete blood counts, hemoglobin, platelet, albumin, blood urea nitrogen (BUN), creatinine, D-dimer, and cholesterol], past medication (antiplatelet drug, anticoagulants, and steroids), complete physical examination and gastroscopy results. Each endoscopist had more than 10 years of experience in endoscopic diagnosis and treatment, and the diagnosis was screened based on the International Classification of Diseases-10. The patients were divided into groups with and without renal decompensation according to whether creatinine was higher than 177 μmol/L (16, 17). Additionally, to better evaluate renal function, we calculated the BUN/creatinine ratio (18).

### Statistical analysis

Mean values with standard deviations (x ± SD) were used to represent quantitative data, and counts with percentages (*n*,%) were used to represent qualitative data. The chi-square test for categorical variables and *t*-tests/Mann-Whitney tests for continuous variables were used to identify univariate associations between the myocardial injury group and the control group. A prediction model was performed based on the significant multiple factors, and a receiver operating characteristic (ROC) curve was used to evaluate the capacity to predict myocardial injury. According to the conclusion of the regression analysis, a nomogram for myocardial injury probability was constructed. Univariate and multivariate logistic regressions were used to select possible factors related to in-hospital outcomes. All data processing and statistical analysis were performed using SPSS version 26.0 (SPSS Inc., Chicago, IL, USA) and R 3.1.2 (The R Foundation for Statistical Computing, Vienna, Austria). Odds ratios (ORs) and 95% confidence intervals (CIs) were calculated, and *P* < 0.05 was considered statistically significant.

## Results

A total of 989 patients (989/1,208, 81.9%) met the inclusion criteria and were analyzed, of whom 101 patients (10.2%) experienced myocardial injury during hospitalization. The general characteristics of all enrolled patients are shown in Table 1.

**TABLE 1:** Clinical characteristics of AUGIB patients with and without myocardial injury.

Patients	In total	Myocardial injury		*P*-value
			
	*N* = 989	With *N* = 101	Without *N* = 888	
Male, *n* (%)	661 (66.8)	60 (59.4)	601 (67.7)	0.094
Age (years)	54.82 ± 15.35	62.36 ± 13.69	53.96 ± 15.30	0.000
Heart rate (bpm)	81.11 ± 14.50	81.23 ± 15.70	81.10 ± 14.37	0.988
SBP (mmHg)	113.59 ± 18.37	118.68 ± 22.83	113.08 ± 17.80	0.121
DBP (mmHg)	68.96 ± 11.84	68.07 ± 14.03	69.04 ± 11.60	0.666
Smoker, *n* (%)	289 (29.2)	29 (28.7)	260 (29.3)	0.906
Drinker, *n* (%)	164 (16.6)	20 (19.8)	144 (16.2)	0.359
**Blood biochemistry**				
Hb (g/L)	91.22 ± 18.34	87.43 ± 19.77	91.65 ± 18.14	0.101
HCT (%)	28.18 ± 5.31	27.14 ± 6.09	28.30 ± 5.21	0.131
WBC (×10^9^/L)	6.56 ± 8.28	6.94 ± 4.52	6.52 ± 8.58	0.000
Platelet (×10^9^/L)	127.27 ± 98.09	129.89 ± 107.32	126.97 ± 97.05	0.970
BUN (mmol/L)	6.90 ± 5.59	10.13 ± 8.82	6.52 ± 4.95	0.000
Creatinine (μmol/L)	74.29 ± 96.58	107.99 ± 119.97	70.34 ± 92.74	0.005
≤177 μmol/L, *n* (%)	914 (92.4)	84 (83.2)	830 (93.5)	0.000
>177 μmol/L, *n* (%)	30 (3.0)	15 (14.9)	15 (1.7)	0.000
BUN/creatinine	105.90 ± 56.65	114.37 ± 62.68	104.91 ± 55.86	0.174
cTNI (pg/mL)	24.03 ± 47.47	48.96 ± 67.21	6.83 ± 6.86	0.015
cTNT (μg/L)	0.03 ± 0.08	0.06 ± 0.11	0.01 ± 0.004	0.000
D-dimer (mg/L)	5.02 ± 7.18	5.33 ± 5.51	4.98 ± 7.36	0.110
Albumin (g/L)	32.94 ± 5.06	32.48 ± 5.25	32.99 ± 5.04	0.744
Cholesterol (mmol/L)	2.90 ± 1.00	2.86 ± 1.11	2.90 ± 0.99	0.592
**History of medicine use**				
Aspirin, *n* (%)	65 (6.6)	11 (10.9)	54 (6.2)	0.071
Anticoagulant, *n* (%)	21 (2.1)	5 (5.0)	16 (1.8)	0.091
Steroids, *n* (%)	19 (1.0)	4 (4.0)	15 (1.7)	0.242
**Comorbidities**				
Hypertension, *n* (%)	145 (14.8)	33 (32.7)	112 (12.8)	0.000
Diabetes, *n* (%)	150 (15.3)	27 (26.7)	123 (14.0)	0.001
Stroke, *n* (%)	54 (5.5)	9 (8.9)	45 (5.1)	0.115
CKD, *n* (%)	41 (4.2)	14 (13.9)	27 (3.1)	0.000
Tumor, *n* (%)	208 (21.3)	26 (25.7)	182 (20.8)	0.246
Digestive system tumor, *n* (%)	187 (18.9)	24 (23.8)	163 (18.6)	0.210
Non-digestive system tumor, *n* (%)	21 (2.1)	2 (1.9)	19 (2.2)	1.000
LVEF <68%, *n* (%)	38 (38.4)	12 (60.0)	26 (32.9)	0.026
Helicobacter pylori, *n* (%)	55 (5.6)	3 (3.0)	52 (5.9)	0.221
**Cause of bleeding**				
Varices, *n* (%)	622 (62.9)	58 (57.4)	564 (63.5)	0.230
Peptic ulcer, *n* (%)	277 (28.0)	28 (27.7)	249 (28.0)	0.946
Others, *n* (%)	77 (7.8)	12 (11.9)	65 (7.3)	0.105
Missing, *n* (%)	13 (1.3)	3 (3.0)	10 (1.1)	0.280
In-hospital death, *n* (%)	29 (2.9)	11 (10.9)	18 (2.0)	0.000

AUGIB, acute upper gastrointestinal bleeding; SBP, systolic blood pressure; DBP, diastolic blood pressure; Hb, hemoglobin; HCT, hematocrit; WBC, white blood cell; BUN, blood urea nitrogen; cTNI, cardiac troponin I; cTNT, cardiac troponin T; CKD, chronic kidney disease; LVEF, left ventricular ejection fraction.

### Baseline characteristics

The study population was mainly male (66.8%), with an average age of 54.82 ± 15.35 years. Compared with the patients without myocardial injury, the patients with myocardial injury tended to be elderly (62.36 ± 13.69 vs. 53.96 ± 15.30, *P* < 0.001) and had significantly higher serum levels of creatinine, BUN, cTNI, cTNT, and white blood cells (WBCs) (all *P* < 0.05). Moreover, there were more patients with a history of hypertension, diabetes, and CKD in the myocardial injury group (all *P* < 0.05). Left ventricular ejection fraction (LVEF) was measured as an indicator of cardiac function and was found to be lower in the myocardial injury group (*P* < 0.05). The patients in the myocardial injury group had a much higher in-hospital mortality rate than those in the control group (10.9 vs. 2.0%, *P* < 0.001). However, a history of tumor was not found to be significantly different between the two groups (*P* = 0.246).

### Risk factors for myocardial injury in patients with acute upper gastrointestinal bleeding

Based on the analysis of the clinical characteristics between the two groups, factors that were used to predict myocardial injury were identified as age, serum level of creatinine, BUN, WBC count, history of hypertension, diabetes, and LVEF (Table 2). All these factors were then assessed using multivariable regression analysis; hypertension (OR: 4.252, 95% CI: 1.149–15.730, *P* = 0.030), BUN (OR: 1.159, 95% CI: 1.026–1.309, *P* = 0.018), and LVEF <68% (OR: 3.667, 95% CI: 1.085–12.398, *P* = 0.037) were found to be independently correlated with myocardial injury in AUGIB patients (Table 2).

**TABLE 2:** Logistic regression analysis of AUGIB patients with myocardial injury.

Characteristics	Multivariate model		
	
	OR	95% CI	*P*-value
Age	1.008	0.960–1.057	0.761
Diabetes	1.225	0.266–5.639	0.795
Hypertension	4.252	1.149–15.730	0.030
WBC	1.054	0.881–1.260	0.568
Creatinine	0.996	0.991–1.002	0.212
BUN	1.159	1.026–1.309	0.018
LVEF <68%	3.667	1.085–12.398	0.037

OR, odd ratio; CI, confidence interval. Other abbreviations as in Table 1.

### Prediction model construction

**FIGURE 2:**
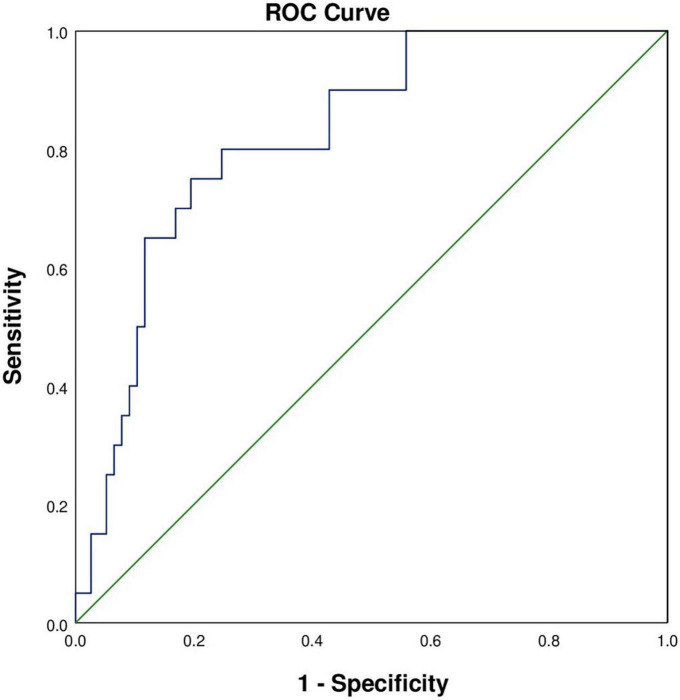
Receiver operating characteristic curve for the identification of patients with myocardial injury. The area under the curve was 0.823 (SD: 0.047, 95% CI: 0.730–0.916).

**FIGURE 3:**
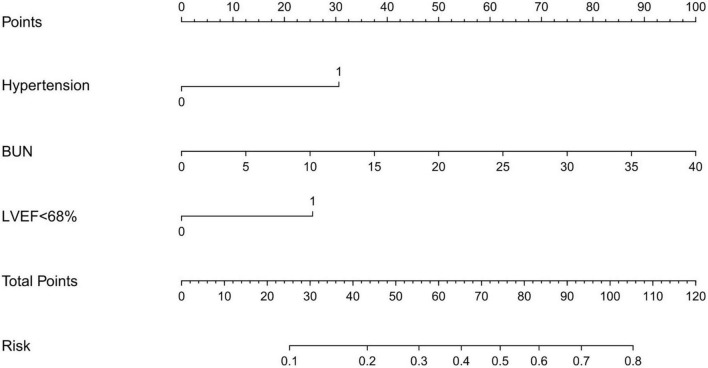
Nomogram for predicting myocardial injury in patients with acute upper gastrointestinal bleeding. BUN, blood urea nitrogen; LVEF, left ventricular ejection fraction.

According to the multivariable regression analysis, a history of hypertension, serum BUN level and LVEF <68% were found to be independent risk factors for myocardial injury. A ROC curve was drawn that incorporated the above three factors with an area under the curve of 0.823 (SD: 0.047, 95% CI: 0.730–0.916), which showed robust discrimination (Figure 2). A nomogram was built for the prediction of myocardial injury after AUGIB (Figure 3). Each value of these variables received a score on the axis of the point scale. The probability of myocardial injury could be estimated by adding each single score to obtain the total score.

### Factors affecting in-hospital outcomes in acute upper gastrointestinal bleeding patients with myocardial injury

To evaluate the risk factors influencing in-hospital outcomes in AUGIB patients with myocardial injury, univariate, and multivariate analyses were performed (Table 3). In the univariate analysis, serum levels of hemoglobin (OR: 0.953, 95% CI: 0.918–0.989, *P* = 0.010), BUN (OR: 1.060, 95% CI: 1.002–1.122, *P* = 0.044), albumin (OR: 0.863, 95% CI: 0.751–0.992, *P* = 0.038), and D-dimer (OR: 1.184, 95% CI: 1.073–1.308, *P* = 0.001) were risk factors for in-hospital death in AUGIB patients with myocardial injury. After adjusting for confounding factors, the serum level of D-dimer was independently correlated with in-hospital death in AUGIB patients with myocardial injury (OR: 1.273, 95% CI: 1.085–1.494; *P* = 0.003).

**TABLE 3:** Factors affecting in-hospital outcomes in AUGIB patients with myocardial injury.

Characteristics	Univariate model		Multivariate model	
		
	OR (95% CI)	*P*-value	OR (95% CI)	*P*-value
Age	0.999 (0.954–1.046)	0.964		
Sex	0.800 (0.227–2.819)	0.728		
Hb	0.953 (0.918–0.989)	0.010	0.947 (0.877–1.023)	0.168
Platelet	0.994 (0.985–1.003)	0.172		
BUN	1.060 (1.002–1.122)	0.044	1.050 (0.967–1.140)	0.246
Creatinine	1.002 (0.997–1.006)	0.423		
Albumin	0.863 (0.751–0.992)	0.038	0.932 (0.731–1.187)	0.568
D-dimer	1.184 (1.073–1.308)	0.001	1.273 (1.085–1.494)	0.003
Diabetes	1.031 (0.253–4.210)	0.966		
Hypertension	0.750 (0.815–3.034)	0.687		
Tumor	2.738 (0.759–9.882)	0.124		

Abbreviations as in Tables 1, 2.

## Discussion

In this single-center, retrospective study, AUGIB patients complicated with myocardial injury were enrolled and analyzed. A history of hypertension, poor renal function, and cardiac function with LVEF <68% were independent risk factors for concomitant myocardial injury. Moreover, AUGIB patients with myocardial injury had an increased death risk in contrast to those with only AUGIB, which was strongly associated with coagulopathy.

AUGIB is an increasingly severe medical emergency due to its high morbidity and mortality rates (2, 19). Although the prognosis of AUGIB patients has improved over the last few decades, the morbidity, and mortality rates remain high. Myocardial injury is one of the most common complications of AUGIB, of which the incidence was found to be up to 10–14%, and is related to increased short- and long-term mortality (20, 21). In our study, the incidence of myocardial injury after AUGIB was 10.2%, which was consistent with previous studies. Due to its high incidence and adverse prognosis, early prediction of myocardial injury in AUGIB patients is essential to help predict prognosis and explore effective treatments.

A history of hypertension has been relevant to an elevated risk of myocardial injury. In our study, the patients with hypertension were at a 4.25 times higher risk of in-hospital myocardial injury than the patients without hypertension. The reason may be that long-term elevated blood pressure increases the pressure on the arterial walls, resulting in injury to the arterial endothelium and creating conditions for the formation of unstable plaque in the blood vessel wall (22). Moreover, high levels of BUN and creatinine have been found to be associated with myocardial injury. A high level of BUN is the manifestation of renal insufficiency, and the degree of oxidative stress and inflammation in the myocardium is significantly higher after impaired renal function and causes myocardial damage (8). Moreover, BUN also reflects acute hemodynamics and neurohumoral changes during acute myocardial infarction (AMI), which might contribute to the identification of high-risk patients (23). We found that there was a significant difference between the patients with renal decompensation in the myocardial injury group and the control group, therefore, renal insufficiency can be confirmed as a risk factor for myocardial injury. In clinical practice, the diagnosis of CKD is mainly based on past medical history, and it is difficult to assess long-term renal function during hospitalization because of the acute onset process of AUGIB. Therefore, to avoid the impact of such incomplete records on the analysis of other factors, a history of CKD was excluded from the multifactorial regression analysis.

We also found that LVEF was highly correlated with myocardial injury, which reflected left ventricular systolic function and was highly correlated with heart failure and cardiomyopathy. Reduced LVEF leads to compensatory activation of the sympathetic nervous system and the renin-angiotensin-aldosterone system (RAAS), which may induce hemodynamic stress change with adverse effects on the myocardium and the circulation (24). Wu et al. (25) observed that AUGIB patients with more than three cardiac risk factors had an increased likelihood of concurrent myocardial injury. Common cardiac risk factors include age, male sex, obesity, and traditional cardiac risk factors, including hypertension, diabetes, hypercholesterolemia, smoking, and first-degree relatives with premature myocardial infarction (male relatives <45 years and female relatives <55 years). Fabio et al. (26) identified elevated troponin as a marker of myocardial injury and identified a variety of associated risk factors, including septic shock, stroke, chronic renal insufficiency, and cirrhosis. Based on earlier research and our study, we concluded that cardiac function with LVEF <68% was one of the predictors of myocardial injury. It should be noted that “normal LVEF” is really not normal in chronic and acute diseases, this notion is similar to that “normal electrocardiogram parameters” are really not normal in these diseases (27). In addition, renal function, age, previous history, and infection status were also found to be associated with myocardial injury. Although most of these factors are immutable, their recognition contributes to reducing associated morbidity and mortality through individualized risk stratification and more aggressive management.

In this study, we developed an intuitive statistical predictive graph based on the above factors, which evaluated the risk of myocardial injury and may help clinicians when propose treatment decisions for AUGIB patients. To our knowledge, this study was the first to build a quantitative nomogram to predict the probability of myocardial injury in AUGIB patients. In our nomogram, the serum level of BUN was the severest contributor to the risk of myocardial injury, followed by hypertension and LVEF <68%. Specific strategies should be used to reduce the risk of myocardial injury, such as ensuring hemodynamic stability, improving cardiac function, and managing blood pressure.

The mechanisms by which myocardial injury develops in patients with AUGIB are multifactorial, including hemodynamic instability caused by reduced circulating blood volume, sympathetic nervous system activation, coagulation activation, etc (28). In addition, cirrhosis is a general reason for variceal upper gastrointestinal bleeding, which leads to several cardiac impairments, including high-output heart failure due to cirrhotic cardiomyopathy, causing a decrease in ventricular responsiveness to physiologic or pharmacologic stress (29). Moreover, AUGIB is always accompanied by systemic inflammation and causes the release of cytokines such as tumor necrosis factor-α and interleukin-1, which could potentiate subclinical heart failure and myocardial dysfunction (30).

In our study, the AUGIB patients who developed myocardial injury had significantly higher in-hospital mortality. High levels of hemoglobin and albumin were found to be protective factors for death in AUGIB patients, while high serum levels of BUN and D-dimer were risk factors. After adjusting for confounding factors, the serum level of D-dimer was independently correlated with in-hospital death in AUGIB patients with myocardial injury. Patients with elevated D-dimer often indicate a high-risk of disseminated intravascular coagulation (DIC) accompanied by coagulation disorder that cannot be excluded (31, 32). Thus, we speculated that coagulopathy may be associated with a poor prognosis in AUGIB patients with myocardial injury. Differently from our study, Sabatine et al. (33) found that anemia is an independent risk factor of major adverse cardiovascular events in patients with ACS, and the severity of anemia affects the prognosis of coronary heart disease. For every 10 g/L decrease in hemoglobin, the mortality rate of patients with cardiovascular events within 30 days increases by 21%. A subgroup analysis of elderly and peptic ulcer patients found that serum albumin level was an independent risk factor of mortality in the overall analysis (34). Elderly patients who had an albumin level greater than 23.5 g/L at admission had a relatively low mortality rate (35).

Myocardial injury was defined as ECG ST-T changes and troponin elevation in the current study, which may be precursors to ACS. In China, AUGIB patients complicated with AMI should undergo gastroscopy before percutaneous coronary intervention (PCI). This strategy has lower mortality and fewer complications compared with a direct PCI strategy. Lin et al. (36) observed that in patients with combined AUGIB and AMI, the endoscopic therapy was most valuable in patients in whom AUGIB was the initial event and those who presented with hemodynamic instability. In patients without these manifestations, the endoscopic therapy can be postponed unless decisions for cardiac management are based on endoscopic findings. In conclusion, for AUGIB patients with AMI as the initial event, the diagnosis and treatment of gastrointestinal endoscopy and coronary intervention should be carefully arranged and should be decided after a joint evaluation with cardiology consultants.

There are some limitations. First, due to differences in the quality of data collection are unavoidable, the factors that may indicate blood loss were not adequately considered. Also, this study did not analyze whether the amount of gastrointestinal bleeding was related to the severity of myocardial injury and the effects of blood transfusion, which led to a lack of evidence on fluid management in AUGIB patients. Second, the results of other baseline data of cardiovascular system, such as coronary angiography (CAG), myocardial magnetic resonance, peripheral arterial ultrasound, and other imaging tests were not included in this study, because the symptoms were generally mild to moderate and transient and did not meet the indications for the relevant tests, also acute gastrointestinal bleeding is often a contraindication to CAG, which may underestimate the incidence of myocardial injury. This study is a retrospective study and continuous troponin measurements during hospitalization were not available for all patients. As a result, we could not explore the relationship between the trends in troponin levels over time and mortality. Despite these limitations, this study may serve as a pavement for future studies on the mechanism of interaction between AUGIB and myocardial injury, allowing a better understanding of AUGIB and providing guidance for its management.

## Conclusion

We identified several clinical factors, such as hypertension, renal dysfunction, and cardiac function with LVEF <68%, that were related to the incidence and prognosis of myocardial injury after AUGIB. The occurrence of myocardial injury may increase all-cause in-hospital mortality in AUGIB patients and coagulopathy was found to contribute to this increased mortality. Therefore, early recognition of ECG-specific patterns and the application of troponin examination should be widely used in clinical care to improve patient outcomes.

## Data availability statement

The raw data supporting the conclusions of this article will be made available by the authors, without undue reservation.

## Ethics statement

The studies involving human participants were reviewed and approved by No. XJTU1AF2022LSK-301. The patients/participants provided their written informed consent to participate in this study.

## Author contributions

YY and TS designed and supervised the study. JH, PD, and XQ drafted the manuscript, obtained informed consents from the patients, and participated in the follow-up of patients. JH, PD, XQ, ZC, GZ, and HL collected the clinical and biochemical data, performed follow-ups, and analyzed the data. All authors contributed to the article and approved the final version for submission.

## References

[1] Laine L Yang H Chang S Datto C. Trends for incidence of hospitalization and death due to GI complications in the United States from 2001 to 2009. *Am J Gastroenterol.* (2012) 107:1190–5. 10.1038/ajg.2012.168 22688850

[2] Kurien M Lobo A. Acute upper gastrointestinal bleeding. *Clin Med.* (2015) 15:481–5. 10.7861/clinmedicine.15-5-481 26430191 PMC4953237

[3] van Leerdam M. Epidemiology of acute upper gastrointestinal bleeding. *Best Pract Res Clin Gastroenterol.* (2008) 22:209–24. 10.1016/j.bpg.2007.10.011 18346679

[4] Khamaysi I Gralnek I. Acute upper gastrointestinal bleeding (UGIB) - initial evaluation and management. *Best Pract Res Clin Gastroenterol.* (2013) 27:633–8. 24160923 10.1016/j.bpg.2013.09.002

[5] Hearnshaw S Logan R Lowe D Travis S Murphy M Palmer K. Acute upper gastrointestinal bleeding in the UK: patient characteristics, diagnoses and outcomes in the 2007 UK audit. *Gut.* (2011) 60:1327–35. 10.1136/gut.2010.228437 21490373

[6] van Hattum E Algra A Lawson J Eikelboom B Moll F Tangelder M. Bleeding increases the risk of ischemic events in patients with peripheral arterial disease. *Circulation.* (2009) 120:1569–76. 10.1161/CIRCULATIONAHA.109.858365 19805650

[7] Cappell M. Gastrointestinal bleeding associated with myocardial infarction. *Gastroenterol Clin N Am.* (2000) 29:423. 10.1016/S0889-8553(05)70121-7 10836188

[8] Chen T Yang Y Wang J Wang J. Curcumin treatment protects against renal ischemia and reperfusion injury-induced cardiac dysfunction and myocardial injury. *Transplant Proc.* (2013) 45:3546–9. 10.1016/j.transproceed.2013.09.006 24314955

[9] Longstreth G. Epidemiology of hospitalization for acute upper gastrointestinal hemorrhage: a population-based study. *Am J Gastroenterol.* (1995) 90:206–10. 7847286

[10] Ruigómez A García Rodríguez L Hasselgren G Johansson S Wallander M. Overall mortality among patients surviving an episode of peptic ulcer bleeding. *J Epidemiol Community Health.* (2000) 54:130–3. 10.1136/jech.54.2.130 10715746 PMC1731615

[11] Lip S Tan L Jeemon P McCallum L Dominiczak A Padmanabhan S. Diastolic blood pressure J-curve phenomenon in a tertiary-care hypertension clinic. *Hypertension.* (2019) 74:767–75. 10.1161/HYPERTENSIONAHA.119.12787 31422693 PMC6756261

[12] Sehested T Carlson N Hansen P Gerds T Charlot M Torp-Pedersen C Reduced risk of gastrointestinal bleeding associated with proton pump inhibitor therapy in patients treated with dual antiplatelet therapy after myocardial infarction. *Eur Heart J.* (2019) 40:1963–70. 10.1093/eurheartj/ehz104 30851041

[13] Nable J Brady W. The evolution of electrocardiographic changes in ST-segment elevation myocardial infarction. *Am J Emerg Med.* (2009) 27:734–46. 10.1016/j.ajem.2008.05.025 19751632

[14] Beyne P Bouvier E Werner P Bourgoin P Logeart D Alliot L Emergency department triage of patients with acute chest pain: definition of cardiac troponin I decisional value to manage patients without electrocardiographic evidence of ischemia. *Clin Chem Lab Med.* (2004) 42:556–9. 10.1515/CCLM.2004.094 15202794

[15] Pagani F Bonetti G Stefini F Cuccia C Panteghini M. Determination of decision limits for ACS:systems cardiac troponin I. *Clin Chem Lab Med.* (2000) 38:1155–7. 10.1515/CCLM.2000.176 11156348

[16] Burgos F Teruel J Herrero J Jiménez M Marcen R Ortuño J [Study of renal function after the administration of a low-osmolarity iodized contrast medium. prospective study]. *Actas Urol Esp.* (1989) 13:94–5. 2728949

[17] Koseoglu S Derin S Huddam B Sahan M. The effect of non-diabetic chronic renal failure on olfactory function. *Eur Ann Otorhinolaryngol Head Neck Dis.* (2017) 134:161–4. 10.1016/j.anorl.2016.04.022 27988196

[18] Ernst A Haynes M Nick T Weiss S. Usefulness of the blood urea nitrogen/creatinine ratio in gastrointestinal bleeding. *Am J Emerg Med.* (1999) 17:70–2. 10.1016/S0735-6757(99)90021-9 9928705

[19] Robertson M Majumdar A Boyapati R Chung W Worland T Terbah R Risk stratification in acute upper GI bleeding: comparison of the AIMS65 score with the glasgow-blatchford and rockall scoring systems. *Gastrointest Endosc.* (2016) 83:1151–60. 10.1016/j.gie.2015.10.021 26515955

[20] Emenike E Srivastava S Amoateng-Adjepong Y al-Kharrat T Zarich S Manthous C. Myocardial infarction complicating gastrointestinal hemorrhage. *Mayo Clin Proc.* (1999) 74:235–41. 10.4065/74.3.235 10089991

[21] Bhatti N Amoateng-Adjepong Y Qamar A Manthous C. Myocardial infarction in critically ill patients presenting with gastrointestinal hemorrhage: retrospective analysis of risks and outcomes. *Chest.* (1998) 114:1137–42. 10.1378/chest.114.4.1137 9792589

[22] Reinstadler S Stiermaier T Eitel C Saad M Metzler B de Waha S Antecedent hypertension and myocardial injury in patients with reperfused ST-elevation myocardial infarction. *J Cardiovasc Magn Reson.* (2016) 18:80. 10.1186/s12968-016-0299-1 27832796 PMC5105316

[23] Richter B Sulzgruber P Koller L Steininger M El-Hamid F Rothgerber D Blood urea nitrogen has additive value beyond estimated glomerular filtration rate for prediction of long-term mortality in patients with acute myocardial infarction. *Eur J Intern Med.* (2019) 59:84–90. 10.1016/j.ejim.2018.07.019 30072202

[24] Hartupee J Mann D. Neurohormonal activation in heart failure with reduced ejection fraction. *Nat Rev Cardiol.* (2017) 14:30–8. 10.1038/nrcardio.2016.163 27708278 PMC5286912

[25] Wu I Yu F Chou J Lin T Chen H Lee C Predictive risk factors for upper gastrointestinal bleeding with simultaneous myocardial injury. *Kaohsiung J Med Sci.* (2007) 23:8–16. 10.1016/S1607-551X(09)70368-7 17282980 PMC11918008

[26] Bellotto F Fagiuoli S Pavei A Gregory S Cati A Silverj E Anemia and ischemia: myocardial injury in patients with gastrointestinal bleeding. *Am J Med.* (2005) 118:548–51. 10.1016/j.amjmed.2005.01.026 15866259

[27] Li H Wang Y Lan P Xie L Zhao Y Lu W Electrocardiographic parameters and prognosis of renal light chain amyloidosis. *Clin Cardiol.* (2020) 43:1160–6. 10.1002/clc.23426 33460229 PMC7534015

[28] Licker M Mariethoz E Costa M Morel D. Cardioprotective effects of acute isovolemic hemodilution in a rat model of transient coronary occlusion. *Crit Care Med.* (2005) 33:2302–8. 10.1097/01.CCM.0000182827.50341.18 16215385

[29] Ma Z Lee S. Cirrhotic cardiomyopathy: getting to the heart of the matter. *Hepatology.* (1996) 24:451–9. 10.1002/hep.510240226 8690419

[30] Mehta N Khan I Gupta V Jani K Gowda R Smith P. Cardiac troponin I predicts myocardial dysfunction and adverse outcome in septic shock. *Int J Cardiol.* (2004) 95:13–7. 10.1016/j.ijcard.2003.02.005 15159032

[31] Lin C Chen Y Chen B Zheng K Luo X Lin F. D-dimer combined with fibrinogen predicts the risk of venous thrombosis in fracture patients. *Emerg Med Int.* (2020) 2020:1930405. 10.1155/2020/1930405 33029403 PMC7530481

[32] Thachil J Lippi G Favaloro EJ. D-Dimer testing: laboratory aspects and current issues. *Methods Mol Biol.* (2017) 1646:91–104. 10.1007/978-1-4939-7196-1_7 28804821

[33] Sabatine M Morrow D Giugliano R Burton P Murphy S McCabe C Association of hemoglobin levels with clinical outcomes in acute coronary syndromes. *Circulation.* (2005) 111:2042–9. 10.1161/01.CIR.0000162477.70955.5F 15824203

[34] Jiménez-Rosales R Valverde-López F Vadillo-Calles F Martínez-Cara J López de Hierro M Redondo-Cerezo E. Inhospital and delayed mortality after upper gastrointestinal bleeding: an analysis of risk factors in a prospective series. *Scand J Gastroenterol.* (2018) 53:714–20. 10.1080/00365521.2018.1454509 29575962

[35] González-González J Monreal-Robles R García-Compean D Paz-Delgadillo J Wah-Suárez M Maldonado-Garza H. Nonvariceal upper gastrointestinal bleeding in elderly people: clinical outcomes and prognostic factors. *J Dig Dis.* (2017) 18:212–21. 10.1111/1751-2980.12459 28205386

[36] Lin S Konstance R Jollis J Fisher D. The utility of upper endoscopy in patients with concomitant upper gastrointestinal bleeding and acute myocardial infarction. *Dig Dis Sci.* (2006) 51:2377–83. 10.1007/s10620-006-9326-7 17151907

